# Supplementary material for: Obstructive Sleep Apnea and Recovery in Athletes: BMI and Neck Circumference and Their Impact on Recovery Capacity and Injury Risk
Source: Life (Basel). 2026 Jan 4;16(1):76. doi: 10.3390/life16010076 (PMC12843434; doi:10.3390/life16010076)
Supplement: Supplementary file 1 [file life-16-00076-s001.zip › table s1.pdf]

Table S1: Detailed search strategies and search results for each database included in the systematic review

Database	Full search strategy	Date searched	Records identified	Records after filters*	Notes
PubMed / MEDLINE	("Obstructive Sleep Apnea"[Mesh] OR "obstructive sleep apnea" OR OSA OR "sleep-disordered breathing") AND (athlete* OR "elite athlete*" OR "professional athlete*" OR sport*) AND (recovery OR regeneration OR performance OR injury) AND ("body mass index" OR BMI OR "neck circumference" OR anthropometr* OR "body composition")	12 May 2025	293	74	MeSH terms and free-text terms combined
Web of Science (Core Collection)	TS=("obstructive sleep apnea" OR OSA OR "sleep-disordered breathing") AND TS=(athlete* OR sport*) AND TS=(recovery OR performance OR injury) AND TS=("body mass index" OR BMI OR "neck circumference" OR anthropometr* OR "body composition")	12 May 2025	144	15	Topic search (title, abstract, keywords)
EBSCO (SPORTDiscus, MEDLINE Complete)	("obstructive sleep apnea" OR OSA OR "sleep-disordered breathing") AND (athlete* OR "elite athlete*" OR sport*) AND (recovery OR performance OR injury) AND ("body mass index" OR BMI OR "neck circumference" OR anthropometr*)	12 May 2025	133	14	SPORTDiscus and MEDLINE Complete databases searched
Google Scholar	"obstructive sleep apnea" athlete recovery BMI neck circumference	12 May 2025	570	13	First 200 results sorted by relevance were manually screened
