# Supplementary material for: Obstructive Sleep Apnea and Recovery in Athletes: BMI and Neck Circumference and Their Impact on Recovery Capacity and Injury Risk
Source: Life (Basel). 2026 Jan 4;16(1):76. doi: 10.3390/life16010076 (PMC12843434; doi:10.3390/life16010076)
Supplement: Supplementary file 1 [file life-16-00076-s001.zip › table s2.pdf]

Supplementary Table S2. Methodological quality and risk of bias assessment of included studies

**Study (First author, year)**	**Title (short)**	**Sport / Population**	**Study design**	**Tool used**	**Key domains assessed**	**Overall risk of bias**	**Main sources of potential bias / limitations**
**Dunican et al., 2019**	Sleep disorders in elite Super Rugby team	Elite rugby union (male)	Cross-sectional observational (in-lab PSG)	**CASP**	Sampling, measurement validity, confounding, reporting	**Low**	Small sample; single-team cohort; limited generalizability; no longitudinal outcomes
**Swinbourne et al., 2016**	Sleep quality & OSA risk factors in athletes	Highly trained team-sport athletes (male & female)	Cross-sectional survey	**CASP**	Sampling, measurement validity, confounding, reporting	**Moderate**	Questionnaire-based screening (PSQI/ESS/OSA risk tools) without objective OSA diagnosis; self-report bias; confounding (training load) not controlled
**Peck et al., 2019**	SDB risk in college football players	NCAA football linemen vs controls	Observational comparative (cross-sectional)	**RoB 2.0** *(non-randomized comparative)*	Group comparability, outcome measurement, confounding, reporting	**Moderate**	Non-randomized group allocation; possible residual confounding (body composition/training); screening-based SDB risk rather than PSG-confirmed OSA
**Dobrosielski et al., 2016**	SDB prevalence in collegiate football	Division I football players (male)	Cross-sectional (screening / prevalence)	**RoB 2.0** *(non-randomized observational)*	Outcome measurement, confounding, selection	**Moderate**	Screening/estimation approach; potential misclassification without full PSG for all; single setting; confounders (sleep schedule, training) limited

Study (First author, year)	Title (short)	Sport / Population	Study design	Tool used	Key domains assessed	Overall risk of bias	Main sources of potential bias / limitations
**Caia et al., 2020**	OSA in professional rugby league	Professional rugby league (male)	Observational exploratory (home PSG)	**Newcastle–Ottawa Scale (NOS)**	Selection, comparability, outcome	**Moderate**	Small sample; positional/ethnic subgroup analyses limited power; single-club sample; limited control of confounders
**Suzuki et al., 2022**	MAD therapy in rugby athletes	Professional rugby athletes (male)	Intervention (MAD), pre–post, non-randomized	**CASP**	Internal validity, confounding, outcome measurement, reporting	**Low**	Small treated subgroup; short intervention duration; no randomization/control group; outcomes mostly short-term
**Suppiah et al., 2021**	Sleep characteristics in elite youth athletes	Elite youth athletes (male & female)	Cross-sectional questionnaire-based (cluster analysis)	**CASP**	Sampling, measurement validity, confounding, reporting	**Moderate**	No objective OSA diagnosis; reliance on PSQI/self-report; possible confounding by school/training schedules
**Surda et al., 2019**	Sleepiness/OSA in elite swimmers	Elite swimmers + comparators	Observational (subset with oximetry/actigraphy)	**CASP**	Measurement validity, sampling, confounding, reporting	**Low**	Objective measures only in a subset; modest sample size; ODI/oximetry (not full PSG) may under/overestimate OSA severity
**Ortiz-Naretto et al., 2020**	Mild OSA in mountaineers at altitude	Amateur mountaineers (male & female)	Prospective field observational (polygraphy)	**NOS**	Selection, comparability, outcome	**Moderate**	Very small sample; extreme setting; limited generalizability; multiple altitude-related confounders

Study (First author, year)	Title (short)	Sport / Population	Study design	Tool used	Key domains assessed	Overall risk of bias	Main sources of potential bias / limitations
**Nabhan et al., 2021**	USOPC screening toolbox	940 elite athletes (Olympic/Paralympic; male & female)	Large observational screening program	**CASP**	Sampling, measurement validity, reporting	**Moderate**	Berlin Questionnaire/PSQI (screening) without PSG confirmation; potential underdiagnosis/misclassification; anthropometrics not objectively measured/reported

Overall risk-of-bias judgments were assigned qualitatively (low, moderate, or high) based on the relative contribution of bias domains rather than numerical scoring. Newcastle–Ottawa Scale (NOS), Critical Appraisal Skills Programme (CASP), and Cochrane Risk of Bias 2.0 tools were applied according to their respective methodological guidelines.
